# The Future of Healthcare–Information Based Medicine

**Published:** 2008-04-15

**Authors:** T Borangiu, V Purcărea

**Affiliations:** * University Politehnica of Bucharest, Faculty of Automatic Control and ComputersRomania; ** ‘Carol Davila’ University of Medicine and Pharmacy, BucharestRomania

**Keywords:** Information based medicine, personalized medicine, information technology, service oriented architectures, clinical genomics, medical imaging, targeted pharmaceuticals, HL7, diagnosis center

## Abstract

The paper discusses how information based medicine has become an increasingly important model of healthcare. Today's patients are better informed and therefore play a more active role in their own healthcare, fuelling the drive towards personalized medicine. Information Based Medicine enables researchers to design targeted therapeutics and rapidly develop best practices guidelines to enable healthcare providers to deliver the most complete individualized healthcare solutions.

Information based medicine is realized thanks to growth in four key areas–*Clinical Genomics, Medical Imaging, Targeted Pharmaceuticals, and Information Systems.*

Also discussed, is how technological advances throughout this decade are changing the discovery, development and delivery of new treatments–with healthcare becoming increasingly personalized as a result. A glimpse into the future of personalised healthcare is presented, highlighting scenarios in development today along with the challenges and perspectives which lie ahead.

## Introduction

Healthcare industry has driven many commendable successes in recent decades. The eradication of smallpox has set the stage for the elimination of other diseases once considered life–threatening, such as tuberculosis and polio. Advances in genetics and biotechnology have enabled scientists understand and treat diseases at a molecular level. Perhaps most striking is the fact that life spans have increased by 40% in the past 50 years. Despite these successes, however, there are still enormous opportunities for improvement. Foremost among these opportunities is a shift towards Information Based Medicine, a model of individualized healthcare whereby patients receive personalized, targeted treatment solutions specific for their individual disease stages as well as their individual genetic and metabolic parameters (6).

The medical domain is considered to be one of the most challenging areas of application in knowledge discovery. Most common approaches focus on providing robust classification systems, which are used to assist the physician in the diagnosis/prognosis process. Medical *data mining* problems possess some unique features which differentiate them from other areas, and make them more difficult to tackle. The main difficulties are related to the complex nature of the data involved (heterogeneous, hierarchical, time series), its quality (possibly many missing values) and quantity. 

Although hospitals hold huge amounts of records belonging to past treated patients, part of this data is not in electronic form, and the idea of transferring it to a database system is usually regarded as time consuming. Also, it is common for the physicians in different hospitals to have slightly different investigation methods. This results in different structures for the data coming from different sources, making it impossible to combine it in most cases. Moreover, since human life is at stake, accurate diagnosis is crucial. Establishing the baseline accuracy for a given dataset is therefore very important as well. Domain knowledge or ethical and social issues are also of great significance. An essential particularity of medical problems is the concept of cost, which is addressed by cost-sensitive classification (4, 5)

Today's population is not just aging, but is also better informed than ever before. The growth of the Internet gives patients access to vast amounts of information that was formerly available only to the medical community. Today's patients play a more active role in their own healthcare and proactively request treatment regimens most appropriate to their individual needs. Better-informed patients will demand healthcare tailored to their individual disease states and delivered with the most state–of–the–art technologies, fueling the drive towards personalized medicine.

## Advances in information technology

The realization of the vision of Information Based Medicine is occurring due to a number of advancements in fields that rely heavily on information technology solutions. Such fields include medical imaging, molecular imaging, and clinical information systems as well as genomics and proteomics. Advances in these fields require corresponding advances in information technology solutions. Not only does information technology provide the technical functionality for research in these areas, but it is also required to transform resulting data into useful, decision–enabling information. Information technology is increasingly being incorporated into all areas of the healthcare industry. From a patient care perspective, the move towards Information Based Medicine requires that data from various sources–such as laboratory tests, genetic tests, medical images, family histories, and patient medical records–be easily stored, integrated, and analyzed. 

The vision for Information Based Medicine centres on the ability to integrate patient–specific information and targeted treatments into the best possible healthcare solution for the patient. Information Based Medicine enables researchers to design targeted therapeutics; it enables multiple players in healthcare and life sciences to develop best practices guidelines; and it enables healthcare providers to deliver the most complete individualized healthcare solutions. Technologies from all areas of medicine provide the foundation for this integrated system, and the information can be seamlessly accessed and amended by researchers and healthcare providers.

The goal of personalized medicine requires that investments and advancements be made on a number of fronts. Growth in four key areas– *Clinical Genomics, Medical Imaging, Targeted Pharmaceuticals, and Information Systems*–is helping to make Information Based Medicine a reality. The development of drugs targeted at specific segments of the patient population will allow for increased efficacy and decreased adverse events. Finally, the creation of information systems to handle the transmission, storage, and retrieval of pertinent medical information will allow physicians to access the most comprehensive medical information about an individual patient and to gain insight from access to population–based data as well. Developments in these four key areas, and others, are enabling the realization of the vision of Information Based Medicine:

- **Clinical Genomics**: A key development for personalized medicine is the adoption of clinical genomics and the integration of genomic information into the medical record. As therapies targeted for patients with specific genotypes are approved, there will be rapid changes as the healthcare industry aggressively adopts clinical genomics technologies.
- **Medical Imaging**: There is a clinical need for the availability of historic images. In order for this implementation to be successful, historic images must be transferred among care providers as the patient moves or changes networks. The transfer of digital information requires systems with a unified set of standards for data entry and storage, like the Digital Imaging and Communications in Medicine (DICOM) standard facilitating information sharing.
- **Targeted Pharmaceuticals**: The development of successful therapeutic agents requires that new drugs be both effective and safe. As the healthcare and life sciences industries continue to move towards personalized medicine, there is a need to stratify patient populations into responders and non-responders for specific therapies. In addition to developing drugs targeted at patients most likely to respond, researchers are also incorporating knowledge into the drug development process to develop therapies targeted at patients least likely to experience adverse reactions.
- **Information Systems**: Appropriate information systems are crucial to enabling Information Based Medicine. Information systems enable biomedical researchers to manage vast data sets to develop targeted treatments. In the future, information systems will enable physicians to incorporate patient–specific data, best practice guidelines, and information about targeted treatments into individualized healthcare solutions.

## Changes in medical science technology and SOA

During the past decade, life sciences and information technology began to converge, resulting in significant and life–impacting research–the result with perhaps the highest impact to date being the sequencing of the human genome and its influence on how clinical researchers now investigate methods and molecules that could improve the human condition. Knowledge gained through human genome sequencing is driving recent achievements in genomic, proteomic, molecular biology and bioinformatics. As this decade progresses, next generation medical science technology and capabilities,enabled by increasingly ‘smarter’ information technology, will change the discovery, development and delivery of new treatments even more dramatically. 

Thus, healthcare will become increasingly personalized as these biologic–based diagnostics and treatments become standard practice. Significant changes in information technology have occurred over the past decade. Recent information technology advances have significantly reduced the cost of storage, enabling the possibility of access to hundreds of biological databases produced by research groups around the world. Storage technology discoveries, high performance computing technologies, advances in digitization technologies and Service Oriented Architectures have given rise to the digitization of patient clinical data (i.e., electronic medical records):

- *Health policy and global spending*: Advances in medical science and medical technology will have limited impact if health policy issues are not simultaneously addressed. Privacy, security, bioethics and bio–discrimination are some of the policy issues to be solved by world governments. Healthcare community efforts that connect stakeholders are becoming more affordable and being adopted by leading hospitals. There is a trend in the healthcare standard communities to give the patient ownership of his or her medical data and thus move the control of the information from the institute to the patient.
- *Progress in science and technology*: Personalized healthcare will be achieved through a composite of scientific advances and new technology, and creative uses of information technology and human thought in the practice of medicine. Scientific advances and discoveries, as well as new technological capabilities, will be revolutionary. Innovation in the practice of medicine will be evolutionary. The combination of revolutionary technologies and evolutionary practices form information based medicine and will shape the future of personalized healthcare. Many industry leaders in pharmacy, academic and medical research centres and hospitals have already taken the first steps toward information–based medicine. By 2010, information–based medicine will change not only the information technology capabilities of these businesses, but their ability to organize, share and utilize knowledge and information across the enterprise and the value chain.
- *Service Oriented Architectures*: Service–Oriented Architecture (SOA) is a design approach that dissolves business applications into separate functions or ‘services’ that can be used independent of the applications and computing platforms (1). In the case of SOA, two types of standards apply: technology standards which govern how services communicate, and industry–specific standards which outline what gets communicated. Healthcare has already progressed to the point of publishing SOA version of its standard (for example Health Level 7–HL7). In order to provide a better integration of the medical investigation equipment and/or medical software produced by various companies, it is absolutely necessary to use the HL7, DICOM, EN 13606 standards, because they give a set of rules and algorithms that are specific to the medical field and especially to the medical information field. The HL7 standard facilitates the communication between various kinds of software applications from the medical information system. HL7 assures the necessary support for message exchanges regarding: patients' management (hospitalization, filling out personal data, medical history, transfer, etc.), scheduling, the patients on the available resources, costs of the medical act, clinical observations, laboratory results, established diagnosis, administrated treatment, and medical document transfer (2,3)

The following is a glimpse into the future of personalized healthcare through scenarios in development today:

**Patient–oriented quality of care**

The first step toward patient oriented quality of care is the development of an infrastructure to support data integration, including information such as electronic patient records, laboratory data, diagnostic imaging, tissue samples and genealogical records–and the algorithms and tools required for analysis. The development of large–scale electronic record keeping systems for the purpose of practicing medicine, including pre symptomatic testing and diagnosis, is a critical element in the evolution of healthcare. These electronic records will be sophisticated heterogeneous objects containing molecular (genotypic) as well as clinical (phenotypic) data.

An example in this respect is the Diagnosis Center–a software application that tackles one of the most important medical issues to date: misdiagnosis. It is comprised, at its essence, of a medical expert system that behaves like an expert doctor. Its purpose is to present steady, unemotional and complete diagnostics in relation to a rule–oriented knowledgebase of disease profiles.

The Diagnosis Center is directed at medical doctors that can use it to enter a patient's condition and find all the possible issues that can generate that condition. This is done by answering a series of questions generated by the application that will lead to the complete list of possible diseases and their corresponding probabilities. 

Usually, Diagnosis Centres revolve around a revolutionary fuzzy medical expert system that is comprised of:

- a medical knowledgebase, based on five types of specialized profiles and an image database.
- a Bayesian inference engine, which uses backward chaining to get to the solution.
- a consensus and validation module, which is able to deal with contradicting hypothesis
- an image processing and pattern matching module, which supports the DICOM (Digital Imaging and Communications in Medicine) standard.
- a knowledge acquisition facility, which gives doctors the chance to develop the knowledgebase of the expert system
- a rich user interface, for both the diagnosis process and knowledge acquisition.

Currently there are a few projects that target the problem of misdiagnosis. Among them we can mention the ‘Inference and advisory system for medical diagnosis’, a project that aims to create a DSS (Decision Support System) for anaemia related diseases, under the supervision of Dr. W.A.J.J. Wiegerinck, and the DSS that is developed for use in the field of haematology and endocrinology, a project supervised by Professor H.J. Kappen. 

**Targeted treatment solutions**

Transparent access to multidimensional heterogeneous data and interoperability of various software applications from multiple sources across drug discovery, development and delivery processes are key capabilities to break current data and communication flow barriers and increase collaboration across the information–based medicine value chain. 

**Advances in medical science and medical technology**

Large–scale population studies are a key tool in the research leading to more individualized treatment solutions. Within the next decade, molecular diagnostic products will likely enable researchers to predict a patient's response to therapy based on the genetic makeup of a tumour (in the case of cancer), the viral genotype (for viral infections) or the genetic make–up of the patient (for a wide variety of conditions).

## Challanges and perspectives for the future

While there are many exciting scientific and medical technologies that promise a new world of medicine, there are some significant challenges to their implementation. The healthcare enterprise is the common denominator in the personalized medicine equation. Unfortunately, devices within the healthcare system do not function as a system today. This creates a number of limitations, including the inability to easily fuse data, share resources, upgrade algorithms and systematically collect data for further analysis. Medicine has not always moved ahead as quickly as technology. Some amount of inertia is useful to provide a necessary level of safety. However, the key issues for the lag–behind include the validation and certification of equipment changes and upgrades, the cost associated with replacing outmoded equipment and the difficulty of intermixing hardware and software from different vendors and generations.

The common thread throughout the information–based medicine ecosystem is the need for knowledge derived from standardized data that can be shared across corporate, agency, physician, provider and consumer boundaries and provided in a safeguarded, compliant manner based upon policies that define the rights and responsibilities for the data being accessed.

In a world that's flat, information is shared almost instantaneously. There is a need to integrate and share medical knowledge and link patients, healthcare providers, and insurers quickly and efficiently through intelligent, information–rich networks. Innovation is all about transforming healthcare infrastructures to provide an integrated view of information through optimized clinical and business processes, health and wellness management, and patient–centric networks. This innovation should lead to vast improvement in the quality of patient care and the economics of healthcare. 
